# TLR4 and TLR9 Expression in Different Phenotypes of Rhinitis

**DOI:** 10.1155/2012/925164

**Published:** 2012-04-10

**Authors:** Maria Lauriello, Alessandra Micera, Paola Muzi, Lino Di Rienzo Businco, Sergio Bonini

**Affiliations:** ^1^Department of Experimental Medicine, University of L'Aquila, Via Vetoio Coppito 2, L'Aquila, Italy; ^2^G.B. Bietti Foundation, IRCCS, Rome, Italy; ^3^Otorhinolaryngology Unit, S. Spirito Hospital, Rome, Italy; ^4^Faculty of Medicine and IFT-CNR, Second University of Naples, 00133 Rome, Italy

## Abstract

*Background*. Toll-like receptors (TLRs) represent a family of evolutionarily conserved proteins, that represent a fundamental link between innate and adaptive immune responses. *Aim*. The purpose of this study was to investigate the expression of TLR4 and TLR9 in the normal nasal mucosa and in the mucosa of subjects with different phenotypes of rhinitis. *Methodology*. A confocal analysis of TLR4 and TLR9 (co)expression was carried out on biopsies from the inferior turbinate obtained from 4 patients affected by persistent allergic rhinitis, 8 patients with chronic rhino-sinusitis, and 6 patients with vasomotor rhinitis The results were compared with those of specimens obtained from 4 subjects undergoing nasal surgery, but with signs of nasal inflammation. *Results*. TLR4 and TLR9 were expressed in the healthy nasal mucosa; TLR4 and TLR9 expression was significantly decreased in allergic rhinitis. TLR4 was over expressed in the epithelium of chronic rhino-sinusitis. Both TLRs were co-expressed in the sub-epithelial infiltrate of chronic and vasomotor rhinitis, even though this expression was higher in the former compared with the latter. *Conclusions*. This study indicates that TLR4 and TLR9 show a different pattern of expression in different phenotypes of rhinitis, possibly related to the type and severity of the disease.

## 1. Introduction

Innate immunity plays a selective and specific role in destroying pathogens and presenting antigens to the cells involved in adaptive immune response [1, 2]. The discrimination between self- and foreign pathogens is mainly mediated by a family of receptors—termed pattern recognition receptors, (PRRs)—which interact with specific ligands of the invading hosts (pathogen-associated molecular proteins (PAMPs)) [1]. PRRs are mainly expressed by epithelial cells, antigen-presenting cells, regulatory T cells, and other cells involved in the interaction between the immune system and the environment [3–5]. Toll-like receptors (TLRs) represent the major family of PRRs—represented at present by 13 members in humans, TLR1 to TLR13, whose localization correlates to some extent with the molecular patterns of their ligands. In particular, TLR1, TLR2, and TLR4 are located on the cellular surface, while TLR3, TLR7 and TLR9 are not expressed on the cell surface as they are involved in the recognition of nucleic-acid-like structures [6]. TLRs activation triggers a cascade of reactions leading to increased expression of proinflammatory genes [7]. As recently reported, TLR signalling affects the development of several human diseases such as bacterial infections, sepsis, immunodeficiencies, and asthma and autoimmune diseases [8].

The aim of the present study was to investigate the expression of TLR4 and TLR9 in the healthy and inflammatory nasal mucosa of different rhinitis phenotypes, by means of confocal analysis on paraffin sections of the inferior turbinates.

## 2. Materials and Methods

### 2.1. Reagents

 All reagents used in this study were of analytical grade and purchased from EuroClone (Milan, Italy) or ICN Biomedicals (Costa Mesa, CA, USA). All sterile plastic ware was from NUNC (Roskilde, Denmark).

### 2.2. Patients and Tissue Sampling

 After informed consent and approval from a local ethic committee, paraffin sections of the inferior turbinate were obtained from 4 patients suffering from seasonal nasal allergy, 8 patients with chronic rhinosinusitis, and 6 patients affected by vasomotor rhinitis,. Normal sections (referred to as control) were obtained from 4 subjects undergoing nasal surgery with no acute or chronic nasal inflammation. Each biopsy was fixed in 10% formalin and routinely processed for basic histology or confocal microscopy.

### 2.3. Confocal Analysis

 Serial sections were prepared according to a routine procedure for histological and immunofluorescent evaluation, including a paraffin inclusion and dewax steps, to provide 5 *μ*m sections. Slides were preincubated (3% bovine serum albumin and 0.5% Triton X-100 in 10 mM in buffered phosphate saline, pH 7.5; PBS) and then incubated overnight at 4°C with a cocktail of the following antibodies: rabbit anti-human TLR4 (4 *μ*g/mL) and goat anti-human TLR9 antibodies (2 *μ*g/mL), purchased from Santa Cruz Biotech (Santa Cruz, CA). Specific binding of the primary antibodies was detected using secondary species-specific Cy32-conjugated anti-rabbit (for TLR4) and Cy3-conjugated anti-goat (for TLR9), all prediluted 1/200 in PBS containing 0.05% Tween-20 and incubated at room temperature for 45 minutes (Jackson ImmunoResearch Laboratories, West Grove, PA). Isotype-matched immunoglobulin G antibodies (control isotype, Vector Laboratories, Burlingame, CA, USA) were incubated in parallel, and the sections were used for channel-series acquisitions. The light source was an argon ion laser (25 mW) giving excitation wavelength in the region 458–584 nm. FITC was excited at 488 nm. Cy3 was excited at 550 nm. Digital images of single optical sections were acquired using a ×60/oil-immersion objective using a E2000U inverted confocal microscope (Nikon, Tokyo, Japan); image sizes were 512 × 512. Negative control cover slides were set by exposing the seriate sections under similar conditions but without the primary antibody. Brightness and contrast levels were evaluated using C1 software (Nikon) and the Adobe Photoshop 7.0 program (Abacus Concepts Inc., Berkeley, CA). 

### 2.4. Integrated Optical Density and Statistical Analysis

 Densitometric analysis was carried out on images collected with the same channel series settings by using the C1 confocal software (Nikon) and then submitted to densitometric analysis using 1D ImageJ software (Image J v1.43; NIH-http://rsb.info.nih.gov/ij/). Single integrated optical density (IntDensity) was registered for each group.

Data are IntDensity values, expressed as the mean ± SD (in the text) and mean ± SEM (in the Figure 5). Parametric ANOVA coupled with Tukey Kramer post hoc analysis was used to detect significant difference (statistical package StatView II for PC; Abacus Concepts Inc., San Jose, CA, USA). A *P* < 0.05 was considered statistically significant. 

## 3. Results

**Figure 1:**
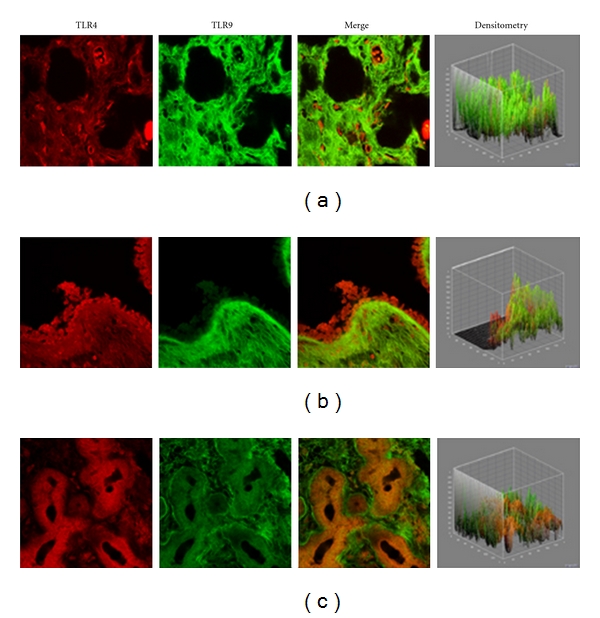
TLR4 and TLR9 expression in healthy turbinates. Confocal analysis showing TLR4 and TLR9 expression at the submucosal layer (a), nasal glands (b), and at the epithelial level (c). Note the strong TLR4 localization at both the epithelial and stromal levels, while TLR9 was localized at the stromal level. ×60/oil-immersion.

In the healthy nasal mucosa, both TLR4 and TLR9 were expressed. As shown in Figures 1(a)–1(c), TLR4 and TLR9 were detected at the submucosal layer (a), nasal gland (b) and epithelial level (c). Particularly, a TLR4 expression (red staining) was mainly localized in the lining epithelial and stromal level (glandular cells), while TLR9 (green staining) was observed in the submucosal tissue (stromal level). 

**Figure 2:**
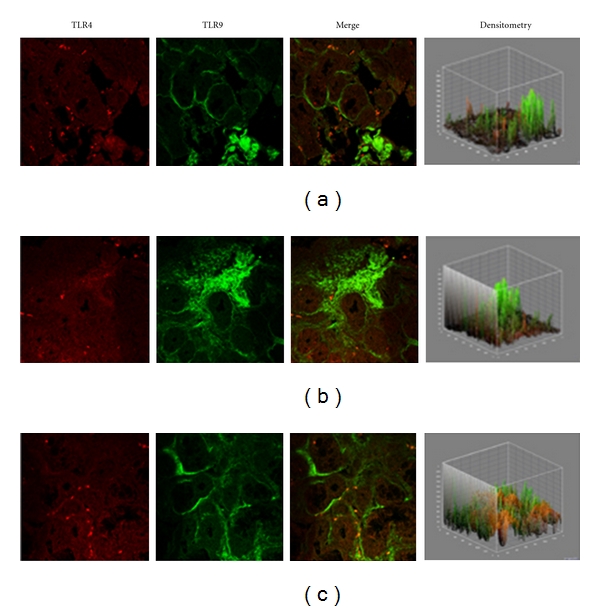
TLR4 and TLR9 expression in allergic rhinitis. Confocal analysis showing a consistent low expression of TLR4 (a, c) and a reduced expression of TLR9 at the stromal level (a) and (b), as indicated by a white arrow in (b) ×60/oil-immersion.

In specimens obtained from the patients suffering by seasonal nasal allergic rhinitis, we detected a lower expression (−47.5% and −48.9%) of both TLR4 and TLR9 proteins at the stromal level, as compared to their healthy control values (201,15 ± 16,61 versus 382,78 ± 60,44; *P* < 0.05 and 219,05 ± 61,65 versus 428,52 ± 116,52; *P* < 0.05; Figures 2(a)–2(c).

**Figure 3:**
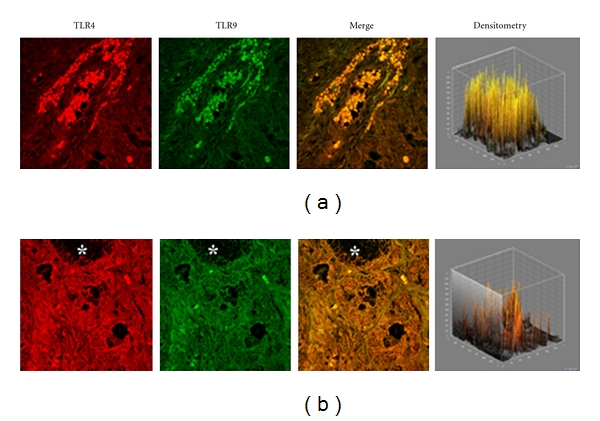
TLR4 and TLR9 expression in chronic rhinitis. Confocal analysis showing the coexpression of TLR4 and TLR9 (a) and (b). Note the strong structural alteration of the glandular epithelium associated with massive cellular infiltrates bearing both TLRs. Asterisk indicates the lumen of a nasal gland. ×60/oil-immersion.

A different pattern of expression was observed in specimens from patients with chronic rhinosinusitis. In fact, while the expression of TLR4 was increased by 40.1% versus that observed in healthy controls (536,45 ± 123,50 versus 382,78 ± 60,44; *P* < 0.05) the expression of TLR9 was decreased by −23,32% (328,60 ± 41,62 versus 428,52 ± 116,52; *P* > 0.05). This pattern of expression (Figures 3(a) and 3(b)) was associated with a marked structural alteration of the glandular epithelium with a massive inflammatory infiltrate showing a straight TLR4/TLR9 colocalization (resulting in a yellow-brown colour in the overlay panel).

**Figure 4:**
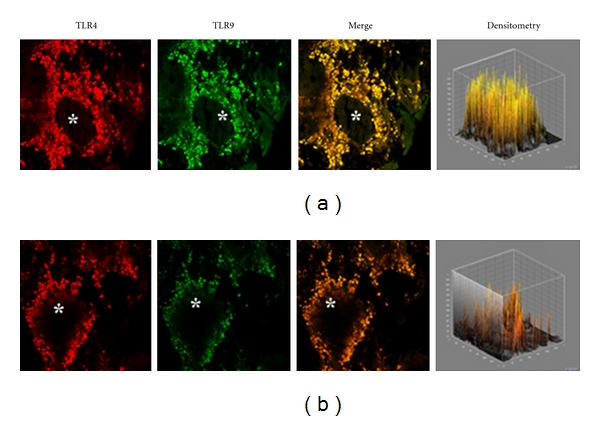
TLR4 and TLR9 expression in vasomotor rhinitis. Confocal analysis showing the coexpression of TLR4 and TLR9 (a) and (b). Note the predominance of the yellow staining indicating the prevalence of the TLR9 expression over TLR4. Asterisk indicates the lumen of a nasal gland. ×60/oil-immersion.

A similar co-localization of TLR4/TLR was also observed in vasomotor rhinitis. However, in this phenotype the expression of both TLR4 (−39.0%) and TLR9 (−63.4%) was significantly downregulated as compared to that of healthy controls (233,51 ± 69,47 versus 382,78 ± 60,44; *P* < 0.05 and 157,06 ± 80,56 versus 428,52 ± 116,52; *P* < 0.05, resp.); Figures 4(a) and 4(b).

**Figure 5:**
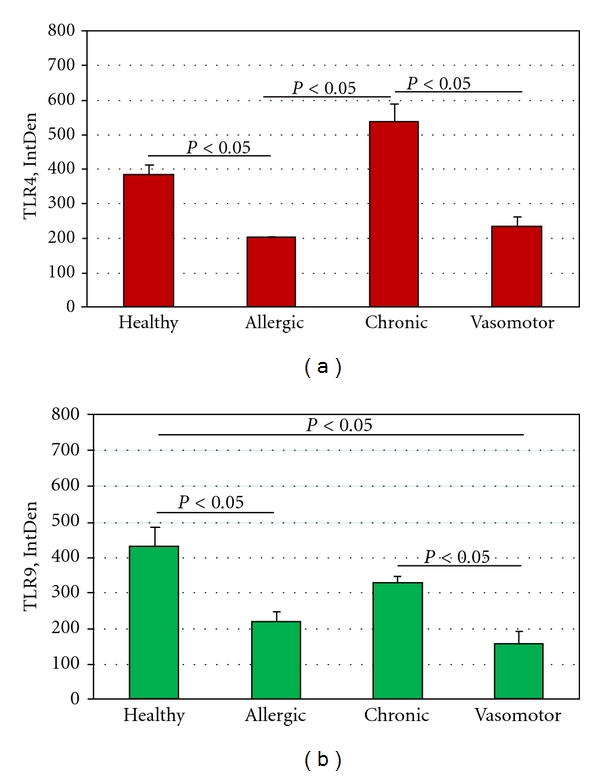
ANOVA analysis of IntDen values among different subgroups.

ANOVA analysis of IntDen values of allergic rhinitis, chronic rhinosinusitis, vasomotor rhinitis and healthy control sections is shown in Figures 5(a) and 5(b).

## 4. Discussion

This study shows that TLR4 and TLR9 are differently expressed in the nasal mucosa of healthy subjects and of the major rhinitis phenotypes. Our findings allow some speculations about the role of TLRs in physiological conditions and in the different inflammatory mechanisms responsible for the different forms of rhinitis.

Both TLR4 and TLR9 are expressed in the healthy nasal mucosa. TLR4 is mainly present in the epithelial layer and in the glandular cells, while TLR9 is mainly detectable at the stromal level. In the submucosal layer, TLR4 is present in scattered cells while the expression of TLR9 is widely diffuse. In the nasal glands, TLR4 was highly expressed while a lower expression was shown for TLR9. The observation of a high TLR4 and TLR9 in the healthy nasal mucosa is consistent with TLR function as the first line of defence against pathogens [7].

Confocal analysis of the inflammatory nasal mucosa in allergic rhinitis revealed a marked downregulation of both TLR4 and TLR9.

TLR4 is mainly expressed by dendritic cells, macrophages and monocytes, as well as by CD3-positive T lymphocytes in the nasal mucosa of young children [9, 10]. It has been suggested that exposure to microbial products in the early life induces a shift of the immune response toward a Th1-type cytokine profile, which protects against the development of allergies and asthma [11]. In fact, exposure to bacterial endotoxin—a ligand for TLR4—was shown to be inversely related to the incidence of atopic asthma, hay fever, and sensitization against aeroallergens in school-aged children [12]. A farming environment is also associated with a lower prevalence of both atopic and nonatopic asthma and an increased exposure to domestic animals implies a decreased atopic risk [13, 14]. LPS/allergen coexposure resulted in the inhibition of allergic inflammation and bronchial hyper-responsiveness, although exposure to LPS after allergen challenge exacerbated the allergic response [15].

Our finding of a lower TLR4 expression in the nasal mucosa allergic rhinitis is in line with the reported low expression of TLR4 in T cells and the lack of response to LPS in atopic adults [10, 16].

Endotoxin-rich environments also contain other immunostimulatory microbial products that act as ligands for TLR2 and TLR9 [17, 18]. While TLR4 is specialized in the recognition of (Gram negative) bacterial products, TLR9 recognizes unmethylated CpG DNA of bacteria and viruses, as well as nucleic acids, which are not unique to the microbial world [2]. Synthetic immunostimulatory sequence oligodeoxynucleotides (ISS-ODNs) activate TLR9 and are more effective than steroids in attenuating the hypersensitivity response of asthma, allergic conjunctivitis, and allergic rhinitis [19, 20]. ISS-ODNs rapidly inhibit the activities of effector Th2 cells and other cells that contribute to allergic response [16]. Interestingly, vaccination with allergen and ISS-ODN and TLR9 ligands has been proposed as a promising approach in immunotherapy of allergic diseases [16, 19–22].

Therefore, our finding of a lower expression of TLR4 and TLR9 in the nasal mucosal of allergic rhinitis patients may be related to the Th2-type allergic inflammation and to the increased susceptibility to upper respiratory infections usually observed in rhinitis patients.

In chronic rhinosinusitis we found that TLR4 is significantly overexpressed. Our data appear in line with previous findings of Dong and coworkers who observed an increased TLR4 expression in chronic rhinosinusitis epithelial cells of inferior turbinate nasal mucosa, as compared to healthy adult volunteers [23]. Accordingly, You and coworkers also reported a significant TLR4 increase in epithelial/glandular cells of chronic rhinosinusitis, compared to nasal polyps and control tissues [24]. On the other hand, a low TLR9 expression has been reported by Ramanatan and coworkers in chronic rhinosinusitis with nasal polyps patients, in comparison to healthy specimens [25, 26].

In rhinosinusitis specimens, confocal analysis showed a clear TLR4/TLR9 co-localization in the cellular infiltrate of the sub-mucosa associated with marked damage of the epithelium. These findings are consistent with the chronic inflammatory state typical of rhinosinusitis.

Although in vasomotor rhinitis both TLR4 and TLR9 appeared to be significantly down-regulated, their co-localization in the cell infiltrate similar to that observed—but in a definitely higher degree—in rhinosinusitis may indicate that a neurogenic minimal inflammation does also occur in this “noninflammatory” rhinitis phenotype [27].

In conclusion, our data indicate that the pattern of expression of TLR4 and TLR is different in healthy subjects and in different forms of rhinitis, possibly in relation to the different pathophysiological mechanisms involved. These findings may contribute to a more accurate phenotyping of rhinitis and may suggest new therapeutic approaches based on the interplay between innate immunity and the adaptive effector response to allergens and microbial agents.
